# Efficacy evaluation of the school program *Unplugged* for drug use prevention among Brazilian adolescents

**DOI:** 10.1186/s12889-016-3877-0

**Published:** 2016-11-29

**Authors:** Zila M. Sanchez, Adriana Sanudo, Solange Andreoni, Daniela Schneider, Ana Paula D. Pereira, Fabrizio Faggiano

**Affiliations:** 1 Department of Preventive Medicine, Centro Brasileiro de Informações sobre Drogas Psicotrópicas, Universidade Federal de São Paulo, Rua Botucatu, 740, 4th floor, 04023-900 São Paulo, SP Brazil; 2 Department of Preventive Medicine, Section of Biostatistics, Universidade Federal de São Paulo, São Paulo, Brazil; 3 Department of Psychology, Universidade Federal de Santa Catarina, Florianópolis, Brazil; 4 Department of Translational Medicine, Avogrado University, Novara, Italy

**Keywords:** Prevention, School, Intervention, Drugs, Alcohol, Adolescents

## Abstract

**Background:**

Most Brazilian schools do not have a continuous program for drug use prevention and do not conduct culturally adapted activities for that purpose. This study evaluated the impact of the *Unplugged* program on drug use prevention among children and adolescents in public middle schools of Brazil.

**Methods:**

A non-randomized controlled trial was conducted in 2013 with 2185 students in 16 public schools from 3 Brazilian cities. The intervention group attended 12 weekly classes of the *Unplugged* program for drug use prevention, and the control group did not attend to any school prevention programs in the same year. Multilevel analyses were used to evaluate temporal and between group changes in the consumption of each drug.

**Results:**

The study suggested that there was no evidence that *Unplugged* effected 11- to 12-year-old students. However, the program seemed to stimulate a decrease in recent marijuana use (transition from use to non-use in 85.7% of intervention cases and 28.6% of control cases, OR = 17.5, *p* = 0.039) among 13- to 15-year-old students. In addition, students in this age range who received the *Unplugged* program had similar drug consumption levels to those observed before the program began. However, students in the control group presented a significant tendency to increase marijuana use and binge drinking.

**Conclusions:**

This study adds to the evidence of program efficacy among Brazilian middle school students by presenting marginal effects on binge drinking and marijuana use. An 18-month randomized controlled trial is recommended for a future study.

## Background

The abuse of alcohol, tobacco, and other drugs by adolescents significantly contributes to the global burden of disease and years of life lost in this age range [1]. The consumption of psychoactive substances during adolescence is growing, making prevention among this age group a basic issue of public health [2]. School is recognized as an adequate setting for the implementation of programs that aim to reduce or delay the start of drug consumption among adolescents because of its universal reach. Nevertheless, a significant part of these programs do not present an evaluation of their effects [3] or, even worse, when evaluated, they do not demonstrate the efficacy and effectiveness in reducing or delaying consumption [4]. There is an international need to improve the availability of evidence-based and culturally adapted prevention programs that target students [5]. Most of the existing prevention programs that target adolescents currently come from the United States [6], which has led the EU-DAP (European Drug Addiction Prevention Project) group to create a program called *Unplugged* [7].

*Unplugged* is a prevention program for adolescents between 11 and 14 years old that aims to delay the onset and interrupt the progression of substance consumption. It was designed to be used by teachers in the classroom in 12 one-hour sessions delivered weekly throughout a school semester. Its structure includes the following themes: social skills, personal skills, knowledge, and normative beliefs [7]. Its effectiveness in reducing consumption has been first evaluated in a wide multicentric study in seven European countries [8], and later it was also evaluated in a smaller study conducted in the Czech Republic [9].

The largest randomized controlled trial carried out in European countries that evaluated the results of the Unplugged program on drug use was conducted on 7079 12- to 14-year-old students and indicated persistent positive effects over 18 months on alcohol abuse and cannabis use but not for cigarette smoking. The program reduced the chances of episodes of drunkenness by 20% (POR = 0.80; 0.67–0.97), the chances of frequent drunkenness by 38% (POR = 0.62; 0.47–0.81) and the chances of recent cannabis use by 26% (POR = 0.74; 0.53–1.00) (Faggiano et al., 2008).

The program is based on the “Comprehensive Social Influence Model” [10], an approach that aims to build specific skills to manage social influences, deconstruct normative beliefs, reflect on the contexts of use, and learn about drugs and their health implications.

Brazil is a country that has a lack of evidence-based drug use prevention programs that target students. Regardless of international evidence that points to school as a highly appropriate setting for the implementation of drug use prevention programs based on the development of social and personal skills (UNODC, 2015), Brazil has never had a public policy to deal with this need. It is important to highlight that the only program that has been systematically implemented in Brazilian schools in the past 3 decades is the DARE - Drug Abuse Resistance Education (Shamblen et al., 2014). The lack of evidence-based prevention programs in Brazilian schools is often attributed to the lack of information and training of the teachers to deal with the subject (Ferreira et al., 2010, Pereira et al., 2016).

The most recent nationally representative data on drug use among public school students showed that 40.3% of adolescents from 13 to 15 years old and 14.1% of 10- to 12-year-old children have used alcohol in the past year. Inhalants and marijuana are the two most prevalent illicit drugs, with a reported past year prevalence of 4.4% and 2.5%, respectively, for the older group and 3.1% and 0.4%, respectively, for the younger group [11]. Due to the worrying data on drug consumption in the country and the mass media pressure to address the so-called “crack epidemic” [12], the Brazilian government has launched the “Integrated Plan to Combat Crack and Other Drugs”, placing drug use as a priority to be addressed by social and collective health policies in the country (Decree 7.637, December 8^th^, 2011).

As part of this initiative, the *Unplugged* program was suggested by the United Nations Office on Drugs and Crime (UNODC) and chosen by the Brazilian Ministry of Health to be adapted for and implemented in Brazilian schools. This paper presents the preliminary results of the effects of the *Unplugged* program to prevent the use of alcohol, tobacco, inhalants, marijuana, cocaine, and crack among 11- to 15-year-old students.

## Methods

### The *Unplugged* program

The *Unplugged* program was delivered by teachers in the classroom. The 12 classes were guided by the student’s and teacher’s manuals and lasted on average 50 min. Both manuals are open access and available in several languages on the website www.eudap.net. The intervention was designed by the EU-DAP group (2009) [7] and includes 4 one-hour classes on attitudes and knowledge about drugs, 4 classes on social and interpersonal skills, and 4 classes on personal skills. In each class, 3 to 5 activities were conducted.

The teachers who delivered the program attended a 16-h training facilitated by coaches who were trained by the European developers, the master trainers of the EU-Dap Intervention Planning Group [9], which means that the program was disseminated by a training cascade from international trainers to local trainers who then trained the teachers selected to carry out the program in the classrooms.

The coaches supervised the implementation at the school. They touched base weekly with the teachers to verify if the class was taught, to ensure that the teachers completed the fidelity check list to control the dose of program delivered (by recording each activity that they implemented and how), and to discuss all of the activities carried out. This coach supervision made this study much more an efficacy trial (high control in the implementation) than an effectiveness trial (real world implementation).

### Study design and population

A quasi-randomized controlled trial was conducted in 16 public schools selected by the State Health Department of three Brazilian cities, São Paulo, São Bernardo do Campo, and Florianópolis (trial registration at the Ministry of Health “Brazilian Register of Clinical Trials - REBEC”, number RBR-2TJRGB). The program allocation was performed at the school level; eight schools received only the usual curriculum (no prevention program) and eight schools received the intervention. All of the schools were in low-income areas to guarantee a similar population of origin. Intervention schools implemented *Unplugged* for 12 weeks from August to November 2013 for the 6^th^ to 9^th^ grade students (between 11 and 15 years old), whereas the control schools did not receive any prevention program in 2013. All of the classrooms for these grades from each selected school were included in the study. It was attested that no other prevention programs would be simultaneously implemented in the schools participating in the study. The baseline assessment of substance use was conducted during the first week of August 2013, and the follow up assessment was carried out 4 months later, during the last week of November 2013 (3 weeks after the end of the program) for both the control and intervention schools.

The sample size of this pilot project resulted from the ability of the Ministry of Health to train and supervise teachers in the three Brazilian cities and from the political agreements between the Ministry of Health and the Regional Education Departments.

### Instrument and measures

The questionnaire used for data collection was an adapted version of that developed and tested by the EU-DAP and used in previous studies on the effectiveness of *Unplugged* [8].

It included modules about the frequency of substance (i.e., alcohol, tobacco, marijuana, inhalants, cocaine, and crack) use in the past month, past year, and lifetime. Alcohol use and binge drinking (i.e., 5 or more doses of alcohol in a single occasion) in the past month was also investigated. For the purpose of this article, the use of substances was dichotomized (i.e., *yes* vs. *no*).

The questionnaires also gathered sociodemographic data and factors associated with drug use (e.g., parental monitoring and support; normative beliefs; knowledge and opinions on drugs; social and personal skills; intention in drug consumption; risk perception; social influence; and school environment).

To pair the questionnaires answered during the two time points of the study (baseline and follow up), students filled in a secret code based on the following information: first name, last name, date of birth, mother’s name, father’s name, paternal grandmother’s name, and eye color. Each code was made of 8 letters and 1 number and could be decoded only by the student themselves, providing them anonymity and confidentiality, which are crucial in a study on illicit behaviors [13]. The secret codes were matched using the Levenshtein algorithm, which identifies similarities among a set of characters.

### Analysis

Descriptive statistics were performed for the past month use of alcohol, tobacco, inhalants, marijuana, cocaine, crack and for the practice of binge drinking.

Due to differences in drug use prevalence between age ranges, the analyses were stratified into two age groups: 11- to 12-year-olds and 13- to 15-year-olds. Due to the hierarchical structure of the data, multilevel analyses were conducted to highlight simultaneous prevalence differences in time and in groups, considering schools as the modeling level (gllamm Stata13) [14]. In each age stratum, the final model (program effect) was adjusted by sex and the baseline prevalence of the investigated drug due to the imbalance of drug use between the intervention and control groups. Due to the low number of cases detected, the insertion of more variables in the multilevel model started to generate unstable and non-converging models. Also considering that the control and intervention schools were selected in the same neighborhoods, the decision was therefore to not include socioeconomic status in the multilevel model.

Considering that the status of drug use can change in two directions (from use to non-use or from non-use to use), we also performed a conditional analysis to investigate the reduction or delay of drug use. This analysis was conditioned to the prevalence of drug use at baseline, and the changes in the two groups were compared. The odds ratio was calculated through a multilevel modelling procedure that adjusted by sex in each age stratum and considered schools as the modeling level (gllamm Stata13).

The analyses were conducted in Stata 13, considering a significance level of 5%.

### Ethical standards

This study was approved by the Ethics in Research Committees at the University of São Paulo (#473.498) and the Federal University of Santa Catarina (#711.377), and all stages of the project were compliant with the Declaration of Helsinki. Consent to participate in the study was obtained from all the schools and subjects.

## Results

### Implementation

All of the intervention schools completed the program. During the intervention, 94% of the planned units were delivered by the teachers (i.e., 698 units were delivered out of the 744 units planned for the 62 groups of students involved in the study). Units not delivered were due to teachers’ absences on the day the class had been scheduled.

### Study participants

**Fig. 1:**
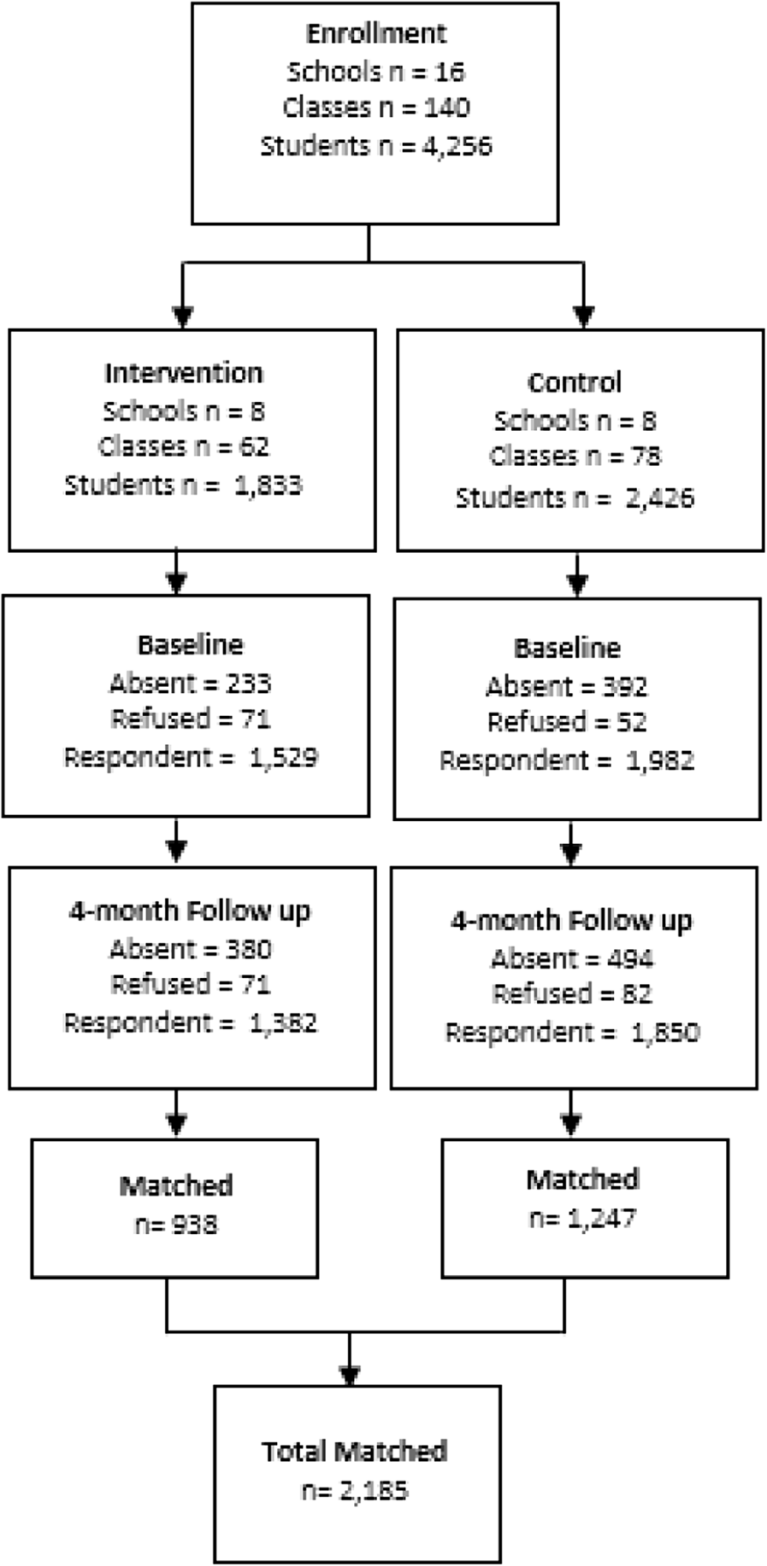
Flowchart of participation in the two groups in the baseline and follow up, Brazil, 2013

Of the 4259 students enrolled in the 140 classes investigated at 16 different schools (average of 9 classes per school), 3511 students answered the baseline questionnaire, and 3232 students answered the follow up questionnaire at the 4-month follow up. Among the participants, 2185 questionnaires were paired at both time points. The differences in participation at the baseline and follow up time points were mainly due to absent students on the day the questionnaires were applied. In Brazil, 30% of students are absent on average in public middle schools [15]. Therefore, considering only the students who were present to respond to the questionnaires at both time points, 85% of the questionnaires were paired (Fig. 1).

Table 1 shows the students’ sociodemographic data distribution. The data showed that there were equal proportions of males and females. There was a higher percentage of students between 13 and 15 years old in the intervention group than in the control group.

**Table 1:** Distribution of the adolescents at the baseline according to sociodemographic variables by study group (*n* = 3511)

	Baseline				Matched			
	Intervention		Control		Intervention		Control	
	(*N* = 1529)		(*N* = 1982)		(*N* = 938)		(*N* = 1247)	
	*N*	%	*N*	%	*N*	%	*N*	%
Gender								
Boys	733	48.8	957	49.1	426	45.7	575	46.6
Girls	768	51.2	993	50.9	506	54.3	659	53.4
Years								
11 – 12	480	34.4	740	40.7	358	40.1	478	41.5
13 – 15	914	65.6	1079	59.3	534	59.9	673	58.5
Grade								
6^th^ grade	364	23.8	507	25.6	228	24.3	312	25.0
7^th^ grade	377	24.7	607	30.6	255	27.2	360	28.9
8^th^ grade	436	28.5	578	29.2	227	24.2	371	29.8
9^th^ grade	352	23.0	290	14.6	228	24.3	204	16.3
State								
SP	951	68.2	1142	62.8	710	75.7	847	67.9
SC	443	31.8	677	37.2	228	24.3	400	32.1

### Baseline prevalence of substance use

There were no significant differences in initial rates of drug use. Alcohol was the most prevalent drug in both ages and groups. At baseline, the prevalence was 7.5 and 5.2% among the younger students and 15.9 and 17.4% among the older students for the intervention and control conditions, respectively.

### Overall intragroup changes from baseline to follow-up

Among the younger students (11-12 years old), there were no significant increases in drug use in both groups during the 4-month follow up. The only significant trend was for alcohol use in the control group. In this group, the prevalence of binge drinking increased from 1.5 to 3.4% (*p* = 0.054).

For the age group of 13- to 15-year-old students, no significant increase in drug use was found in the intervention group from baseline to follow up. However, in the control group, there was a statistically significant increase in past month binge drinking (from 10.6 to 14.9%; *p* = 0.014) and marijuana use (from 1.3 to 2.7%; *p* = 0.050).

### Program effects at the 4-month follow-up

In both age strata, no differences were found in past month drug prevalence from baseline to the follow up in either group (i.e., intervention and control). Additionally, no differences were found when multilevel modeling was performed to compare the transitions in time between groups and to evaluate the program effect, as presented in Table 2.

**Table 2:** Distribution of past month drug use by age according to group and time point

11–12 years	Intervention						Control						Program Effect ^a^	
	Baseline		4 month follow-up		OR^b^ (95% CI)	*p*	Baseline		4 month follow-up		OR^b^ (95% CI)	*p*	OR^a^ (95% CI)	*p*
	*N*	%	*N*	%			*N*	%	*N*	%				
Tobacco	1/296	0.3	3/296	1.0	Non-estimable		0/384	0.0	0/384	0.0	Non-estimable		Non-estimable	
Alcohol	19/251	7.6	19/251	7.6	1.00 (0.32;3.14)	. > 0.999	18/349	5.2	20/349	5.7	1.45 (0.43;4.83)	0.529	0.67 (0.12;3.77)	0.646
Binge drinking	12/226	5.3	15/226	6.6	1.81 (0.51;6.44)	0.345	5/325	1.5	11/325	3.4	6.33 (0.91; 43.95	0.054	0.25 (0.17;1.54)	0.327
Marihuana	0/249	0.0	2/249	0.8	Non-estimable		1/378	0.3	4/378	1.1	Non-estimable		Non-estimable	
Inhalants	2/246	0.8	4/246	1.6	2.50 (0.34;18.30)	0.367	1/378	0.3	5/378	1.3	6.63 (0.62;70.63)	0.117	0.38 (0.02;7.73)	0.527
Cocaine	0/246	0.0	1/246	0.4	Non-estimable		0/377	0.0	0/377	0.0	Non-estimable		Non-estimable	
Crack	0/246	0.0	0/246	0.0	Non-estimable		0/377	0.0	0/377	0.0	Non-estimable		Non-estimable	
13–15 years														
Tobacco	11/441	2.5	15/441	3.4	2.10 (0.62;7.20)	0.235	17/547	3.1	17/547	3.1	1.00 (0.32;3.11)	>0.99	1.45 (0.34;6.25)	0.615
Alcohol	59/370	15.9	71/370	19.2	1.52 (0.90;2.56)	0.116	80/460	17.4	88/460	19.1	1.26 (0.79;2.01)	0.341	1.38 (0.76;2.50)	0.289
Binge drinking	40/340	11.8	38/340	11.2	0.89 (0.47;1.71)	0.742	45/424	10.6	63/424	14.9	2.05 (1.16;3.63)	0.014	0.43 (0.31;1.46)	0.581
Marihuana	7/432	1.6	13/432	3.0	2.41 (0.79;7.33)	0.120	7/546	1.3	15/546	2.7	3.21 (1.00;10.28)	0.050	0.75 (0.16;3.61)	0.722
Inhalants	10/430	2.3	7/430	1.6	0.61 (0.19;1.91)	0.393	10/540	1.9	13/540	2.4	1.46 (0.54;3.96)	0.455	0.41 (0.09;1.91)	0.259
Cocaine	0/431	0.0	5/431	1.2	Non-estimable		1/544	0.2	5/544	0.9	11.96 0.55;259.45)	0.114	Non-estimable	
Crack	0/433	0.0	6/433	1.4	Non-estimable		1/545	0.2	5/545	0.9	11.49 (0.57;230.50)	0.111	Non-estimable	

^a^OR for the multilevel analysis gllamm - intervention *x* control at 4 month follow-up adjusted for sex and baseline prevalence of drug use

^b^ OR for the multilevel analysis gllamm, 4 month follow up *x* baseline, in each group (or intervention or control), adjusted by sex

Considering the conditional analysis (Table 3), the type of transition from baseline was not significant for any measure of the younger age group because of the very low prevalence of students transitioning in both surveys.

**Table 3:** Probabilities of transition between drug use in the past month, considering the changes over time

11–12 years	Transition from baseline to follow-up	Intervention		Control				
		*N*	%	*N*	%	OR	95%CI	*p*
Tobacco	non-use to use	2/295	0.7	0/384	0.0	Non-estimable	Non-estimable	Non-estimable
	use to non-use	0/1	0.0	0/0	0.0			
Alcohol	non-use to use	8/232	3.4	7/331	2.1	1.77	0.46;6.83	0.409
	use to non-use	8/19	42.1	5/18	27.8	2.49	0.45;13.73	0.296
Binge drinking	non-use to use	8/214	3.7	8/320	2.5	1.53	0.50;4.68	0.453
	use to non-use	5/12	41.7	2/5	40.0	0.96	0.10;9.04	0.971
Marihuana	non-use to use	2/249	0.8	4/377	1.1	Non-estimable	Non-estimable	Non-estimable
	use to non-use	0/0	0.0	1/1	100.0			
Inhalants	non-use to use	3/244	1.2	5/377	1.3	Non-estimable	Non-estimable	Non-estimable
	use to non-use	1/2	50.0	1/1	100.0			
13–15 years								
Tobacco	non-use to use	11/430	2.6	8/530	1.5	1.70	0.68;4.26	0.259
	use to non-use	7/11	63.6	8/17	47.1	2.02	0.42;9.62	0.378
Alcohol	non-use to use	38/311	12.2	36/380	9.5	1.39	0.80;2.42	0.241
	use to non-use	26/59	44.1	28/80	35.0	1.39	0.65;2.94	0.393
Binge drinking	non-use to use	17/300	5.7	36/379	9.5	0.57	0.31;1.08	0.084
	use to non-use	19/40	47.5	18/45	40.0	1.28	0.53;3.12	0.583
Marihuana	non-use to use	12/425	2.8	10/539	1.9	1.53	0.66;3.59	0.323
	use to non-use	6/7	85.7	2/7	28.6	17.49	1.16;63.21	0.039
Inhalants	non-use to use	5/420	1.2	9/530	1.7	0.70	0.23;2.11	0.528
	use to non-use	8/10	80.0	6/10	60.0	2.56	0.34;19.18	0.358

*cocaine and crack have been excluded from this table because only 1 transition has been observed in the use of each substance

However, in the age group of 13- to 15-year-olds, some effect was apparent; among non-users, there was a small but not statistically significant increasing trend for tobacco, alcohol and marijuana use from baseline to follow up in the intervention group. The opposite trend appeared for inhalants. For binge drinking, a significant decreasing trend was found for older students transitioning from non-use to use in the intervention group compared with that found for those in the control group. Among users at baseline, trends appeared to always be in favor of the intervention group; there was a statistically significant transition from use to non-use of marijuana in the intervention group compared with that in the control group.

### Attrition

As expected, students who missed the 4-month follow up showed a significantly higher prevalence in the use of some substances evaluated at baseline. Among the 11- to 12-year-old students, there was a higher percentage of paired students (present at baseline and follow up) in the intervention group (343/385 = 41.1%) than in the control group (131/378 = 34.7%), *p* = 0.034. Among the 13- to 15-year-old students, there was a higher proportion of drug use among non-paired students than among paired students for all substances evaluated. However, the differences were significant for only past month alcohol (19.8% vs 24.3%; *p* = 0.029), marijuana (1.6% vs 3.8%; *p* = 0.005) and inhalant (2.2% vs 4.4%; *p* = 0.014) use.

## Discussion

The objective of this study was to evaluate the effect of an international school-based intervention on the prevention of substance use and to evaluate the suitability of its adaptation to the Brazilian culture. This study suggested that students who received the *Unplugged* program in the classroom had similar drug consumption levels to those observed before the program began. However, students in the control group tended to increase their recent marijuana use and practice of binge drinking, suggesting that the program inhibited the expected increase in drug consumption among 13- to 15-year-old students. In this sample, the *Unplugged* program seemed to stimulate a change in marijuana use status because a higher proportion of students in the intervention group reported a transition from recent marijuana use to non-use. However, it is important to note that we dealt with a small number of cases due to the low prevalence of drug use among Brazilian students. In addition, there was a significant reduction in the practice of binge drinking in the intervention group compared with that in the control group.

Nevertheless, the program showed no effect among the 11-12 year old group for two main reasons: the extremely low prevalence of substance use in this age group, i.e., less than 1% for drugs and less than 3% for smoking, and the low effectiveness of *Unplugged* among early adolescents as shown in a previous study [16]. The low prevalence of substance use in Brazil was confirmed by recent survey data [11] that showed the national prevalence of past year tobacco, alcohol, marijuana, cocaine and crack use to be 1.9, 14.1, 0.4, 0.2 and 0.1%, respectively, among those aged 10-12 years.

Adolescence is the period in life in which people begin the consumption of substances. This period is characterized by a continuous, annual increase in consumption until the consumption of all drugs reaches a peak incidence and prevalence around age 18 [17]. Thus, school is the most appropriate environment for primary prevention as the majority of adolescents are enrolled in schools and can be reached by the program [18]. As observed in this Brazilian study, the *Unplugged* program seems to stimulate a decrease in marijuana use among 13- to 15-year-old students. It also seems to delay the expected growth in consumption, given that the control group presented an increase in consumption and the intervention group presented stable marijuana, alcohol, and inhalant use and binge drinking. Despite the marginal results, the program was a success because all classes concluded the program in the expected time without any schools dropping out (Medeiros et al., 2016).

A reduction in marijuana use after *Unplugged* implementation was previously found by Gabrhelik et al. (2012) [9]. Adolescent marijuana use is a compromising behavior because this drug causes episodic impairments and abnormal hippocampus morphology, which may compromise the academic performance of students who consume it [19], making *Unplugged* an interesting program in the school context.

Another interesting result is related to the difference in the progression of binge drinking practices among adolescents in the intervention and control groups. This type of alcohol consumption has been highlighted as one of the main risk behaviors among Brazilian adolescents; it is highly prevalent across different social classes, although the highest risk levels are among the most privileged social classes [20]. Furthermore, a delay in the start of alcohol consumption seems to protect students from practicing binge drinking during adolescence [21] and from alcohol dependence in the future [22].

The fact that the implementation of the program was supported by the Ministry of Health and by the city and state level Departments of Education has certainly contributed to the high fidelity and, consequently, the high dose of the program for students in the intervention group (Medeiros et al., 2016). In the European countries that participated in the randomized controlled trial of *Unplugged* in 2006, 56% of the groups completed the program [8], while in Brazil, this percentage reached 92%.

The process evaluation of Unplugged in Brazil suggested that the dose of the program offered in the classrooms was satisfactory, as almost all the classrooms received 12 lessons. However, the number of activities completed in each class was inadequate; almost half of the lessons were not completed during the 45- to 50-min class period. Additionally, teachers, students, stakeholders, school administrators and the program coaches perceived positive results in the school environment, and teachers and students mentioned an improvement in their relationship. However, teachers had to deliver the Unplugged lessons during their regular class time and did not have additional hours to teach the subjects that were replaced by the Unplugged activities; thus, an adaptation is needed to allow Unplugged to become a part of the Brazilian curriculum (Medeiros et al., 2016).

It is known that a significant portion of these school program interventions are not evaluated or, when evaluated, they are not successful at reducing, delaying, or avoiding the increase in drug consumption among adolescents [4, 23, 24], generating disbelief in the implementation of such school programs [25]. Thus, the *Unplugged* program becomes an option because it has shown potential effects when applied to Brazilian adolescents at the start of the epidemic curve for alcohol and tobacco consumption [11].

It is important to point out the limitations of this study. The first limitation is related to the quasi-experimental design of the study, which may be responsible for the baseline differences in the prevalence of drug consumption among the groups. Education departments selected intervention schools based on their good relationship with school principals, which led to the easy inclusion of *Unplugged* in their 2013 curricula. Nonetheless, such imbalance in the prevalence of drug consumption among groups at baseline was also observed in the first randomized study of *Unplugged* in Europe [8]. Moreover, a 4-month follow up may not be enough to identify strong changes in drug use among Brazilian students.

Another potential limitation of this study was the excessive number of absent students at baseline and follow up, as previously described in a national survey [15]. Nevertheless, no difference in attrition was observed between the control and intervention groups.

The strengths of the study must be highlighted. First, the selection of control and intervention groups was made at the school level and not at the classroom level, avoiding contamination between groups. Second, the control and experimental schools were paired by neighborhood to allow for more homogeneity in terms of students’ socioeconomic status and social factors. Also, there was high fidelity of the program implementation, such that almost all of the classes were taught to all groups. Finally, this study is the first controlled trial conducted in Brazilian schools to test the impact of a prevention program that used standardized techniques in 3 cities and that implemented a rigorous weekly program controlled by coaches, making this an innovative study.

## Conclusion

This study showed that Unplugged had a marginal effect on 13- to 15-year-old students and no effect on 11- to 12-year-old students in reducing past month drug use. A larger and longer randomized controlled trial concentrating on older adolescents is needed to establish the efficacy and cost-effectiveness of the program in Brazilian population. No iatrogenic effects were found, but a longer follow up is needed to establish drug use changes throughout the year.

## Abbreviations

EU-DAP: European Drug Addiction Prevention Project
REBEC: Brazilian Register of Clinical Trials
UNODC: United Nations Office on Drugs and Crime
